# Naringin Reverses Hepatocyte Apoptosis and Oxidative Stress Associated with HIV-1 Nucleotide Reverse Transcriptase Inhibitors-Induced Metabolic Complications

**DOI:** 10.3390/nu7125540

**Published:** 2015-12-10

**Authors:** Oluwafeyisetan O. Adebiyi, Olubunmi A. Adebiyi, Peter M. O. Owira

**Affiliations:** Department of Pharmacology, Discipline of Pharmaceutical Sciences, School of Health Sciences, University of KwaZulu-Natal, Westville Campus, Durban 4001, South Africa; fadebiyi@gmail.com (O.O.A.); olubunmide@gmail.com (O.A.A.)

**Keywords:** naringin, NRTIs, metabolic complications, apoptosis, oxidative stress

## Abstract

Nucleoside Reverse Transcriptase Inhibitors (NRTIs) have not only improved therapeutic outcomes in the treatment of HIV infection but have also led to an increase in associated metabolic complications of NRTIs. Naringin’s effects in mitigating NRTI-induced complications were investigated in this study. Wistar rats, randomly allotted into seven groups (*n* = 7) were orally treated daily for 56 days with 100 mg/kg zidovudine (AZT) (groups I, II III), 50 mg/kg stavudine (d4T) (groups IV, V, VI) and 3 mL/kg of distilled water (group VII). Additionally, rats in groups II and V were similarly treated with 50 mg/kg naringin, while groups III and VI were treated with 45 mg/kg vitamin E. AZT or d4T treatment significantly reduced body weight and plasma high density lipoprotein concentrations but increased liver weights, plasma triglycerides and total cholesterol compared to controls, respectively. Furthermore, AZT or d4T treatment significantly increased oxidative stress, adiposity index and expression of Bax protein, but reduced Bcl-2 protein expression compared to controls, respectively. However, either naringin or vitamin E significantly mitigated AZT- or d4T-induced weight loss, dyslipidemia, oxidative stress and hepatocyte apoptosis compared to AZT- or d4T-only treated rats. Our results suggest that naringin reverses metabolic complications associated with NRTIs by ameliorating oxidative stress and apoptosis. This implies that naringin supplements could mitigate lipodystrophy and dyslipidemia associated with NRTI therapy.

## 1. Introduction

The introduction of highly active antiretroviral therapy (HAART) has reduced the morbidity and mortality associated with human immunodeficiency virus (HIV) infections [1,2,3]. Drug classified as nucleoside or nucleotide reverse transcriptase inhibitors (NRTIs or NtRTIs), non-nucleoside reverse transcriptase inhibitors (NNRTIs), protease inhibitors (PIs), integrase inhibitors and fusion/entry inhibitors are traditionally used in the management of HIV infections [4,5]. The current guidelines on administration of HAART recommend a combination of two NRTIs, one NNRTIs or a protease/integrase inhibitor depending on efficacy and the patient’s tolerability [4,5]. NRTIs (abacavir, didanosine, lamivudine, stavudine, zidovudine and emtricitabine) act as false substrates that sabotage viral cDNA chain elongation hence inhibiting viral reverse transcriptase activity and consequently limiting viral replication [4].

Zidovudine (AZT) and stavudine (d4T) have historically been included as components of various combinations of NRTIs which serve as backbone of HAART [6]. While AZT has remained important in the prevention of mother-to-child transmission of HIV, d4T has remained relevant in the economically less privileged countries because of its relative affordability compared to the preferred alternatives [7,8,9,10]. High incidences of metabolic side-effects such as lipodystrophy, metabolic syndrome, peripheral neuropathy, myelosuppression, hepatic steatosis and lactic acidosis have been reported in patients using NRTIs [11,12,13,14,15]. Therefore, while antiretroviral agents have reduced the morbidity and mortality associated with HIV infection, there is persistent increase in the prevalence of these metabolic complications which threaten the success obtained so far with HAART treatment.

NRTIs are associated with hepatotoxicities such as, steatosis, steatohepatitis, disorders of lipid regulation, hepatic enlargement and abnormal liver functions, [16,17]. Furthermore, the World Health Organization (WHO) has advocated the phasing out of d4T from the available list of antiretrovirals due to severe hyperlactatemialactic acidosis and hepatotoxicity, compared to other NRTIs [12,16,17,18].

Although specific mechanisms through which these complications of NRTIs occur are yet to be clearly defined, it has so far been shown that NRTIs inhibit DNA polymerase gamma thereby leading to a depletion of the mitochondrial DNA and subsequently mitochondrial toxicity [19]. This leads to impaired oxidative phosphorylation (OXPHOS) and subsequent oxidative damage to the cellular machinery coupled with a delay in cell cycle progression which eventually result in apoptotic cell death [12]. These effects have been attributed to the binding of NRTI-triphosphates (the active metabolite of most NRTI following intracellular phosphorylation) to the replicating mitochondrial DNA causing termination of the viral chain elongation [19,20]. Marked increase in reactive oxygen species (ROS), malondialdehyde (MDA, an end-product of lipid peroxidation), and carbonyl proteins (an end-product of protein oxidation), coupled with a decrease in the activities of the enzymatic antioxidant proteins consequent upon a disorder in the oxidative phosphorylation process, have been associated with NRTI administration [16,21].

Currently, there are no standard treatment guidelines for these non-progressive but permanent metabolic complications. Withdrawal from and switching of antiretroviral drug regimens, adjunct pharmacotherapy, and surgical interventions, have previously been tried with limited success [15]. Dietary and nutritional therapies have remained viable options that have not been vigorously pursued. Beneficial effects of some currently available antioxidants have been demonstrated using animal models, but are yet to be validated with large-scale clinical trials [22,23]. There is therefore a need to screen drugs with proven antioxidant effects in the management of the attendant complications of NRTIs.

Plant-derived flavonoids such as naringin (4′,5,7-trihydroxyflavone 7-rhamnoglucoside) which are commonly found in citrus fruits have been recommended as beneficial in reducing the risk of diabetes and cardiovascular diseases in predisposed populations [24]. Its free radical scavenging and antioxidant, anti-apoptotic, antihyperglycemic, antimutagenic, anticancer, anti-inflammatory and cholesterol lowering potentials have been demonstrated [25,26]. Since HIV itself causes symptoms which are similar to those of NRTI-induced metabolic complications [22], it becomes cumbersome to differentiate between the effect of any form of intervention on either the NRTIs administered or on the viral pathogenesis. In this study, we created a model of NRTI-induced metabolic complications in the absence of the HIV infection in order to clearly delineate the observed effects of naringin. The present study was designed to investigate the potential of naringin in reversing metabolic complications of NRTIs and to identify possible mechanisms underlying these observed activities of naringin.

## 2. Materials and Methods

### 2.1. Experimental Animals

Eight weeks old, male albino Wistar rats (200–250 g) were purchased from and housed within the premises of the Biomedical Resources Unit (BRU) of the University of KwaZulu-Natal, Durban, South Africa. They were placed in well-ventilated standard plastic cages, exposed to 12:12-h light-dark cycle at an ambient temperature of 23 ± 2 °C and humidity of 55% ± 5%. Animals were allowed free access to tap water and were fed with standard rat chow *ad libitum*. Ethical approval for this study was obtained from the Animal Ethics Committee of the University of KwaZulu-Natal (reference number: 008/14/animal) and the animals were handled humanely in accordance with the guidelines provided by the same body.

### 2.2. Drugs and Chemicals

Naringin, butylated hydroxytoluene (BHT), thiobarbituric acid (TBA), trichloroacetic acid (TCA), guanidine, ethanol, ethyl acetate, 2, 4-dinitrophenylhydrazine (DNPH), phosphoric acid (H_3_PO_4_), hydrochloric acid (HCl) were purchased from Sigma-Aldrich^®^ chemicals, St. Louis, MT, USA. d4T and AZT from Aspen Pharmacare^®^, Durban, South Africa, while vitamin E was purchased from PharmaNatura (Pty) LTD Sandton, South Africa.

### 2.3. Experimental Design

The rats were divided into seven groups (*n* = 7), (Table 1). All drugs were dissolved in distilled water, which served as the vehicle, prior to administration. Rats in groups I, II and III were treated daily with 100 mg/kg body weight (BW) of AZT by oral gavage [27,28], while groups IV, V and VI were similarly treated with 50 mg/kg BW of d4T [29]. Additionally, rats were treated orally with 50 mg/kg BW of naringin (groups II and V) [30] and 45 mg/kg BW of vitamin E, which was served as the positive control in the study, (groups III and VI) [31], respectively. Rats in group VII served as the vehicle-treated control and were given 3 mL/kg BW of distilled water by oral gavage.

On the 56th day of treatment, rats were sacrificed by halothane overdose, blood was collected by cardiac puncture, centrifuged at 3000 rpm for 10 min and plasma samples stored at −80 °C for further biochemical analysis. Liver as well as visceral and mesenteric fat were promptly surgically removed for further analysis.

**Table 1:** Animal treatment schedule.

Groups	Treatment (Dose; mg/kg/Day)			
	AZT	d4T	Naringin	Vitamin E
I	100			
II	100		50	
III	100			45
IV		50		
V		50	50	
VI		50		45
VII	Distilled Water (3 mL/kg/body weight/day)			

AZT: (zidovudine); d4T: (stavudine).

### 2.4. Biochemical Analysis

#### 2.4.1. Fasting Plasma Lipid Profile Estimation

Fasting plasma Total Cholesterol (TC), High Density Lipoprotein Cholesterol (HDL) and Triglycerides (TG) were measured by the Olympus AU 600 auto analyzer (Alternative Biomedical Solutions, Dallas, TX, USA).

#### 2.4.2. Liver Thiobarbituric Acid Reactive Substances (TBARS) Assay

TBARS assay was carried out following the modified method of Halliwell and Chirico [32]. Briefly, 100 mg of liver tissues were homogenized in 500 μL of ice-cold 0.2% H_3_PO_4_ solution and spun at 1600× *g* for 5 min at 4 °C. Subsequently, 200 μL of the supernatant were added to 500 μL of 2% H_3_PO_4_, 400 μL of 7% H_3_PO_4_ and 400 μL of BHT/TBA solutions in a set of clean glass test-tubes, respectively. In another set of eight clean fresh test tubes, 200 μL of serially diluted MDA standard was added to 500 μL of 2% H_3_PO_4_, 400 μL of 7% H_3_PO_4_ and 400 μL of BHT/TBA solutions, respectively. Reactions in both sets of tubes were initiated with 200 μL of 1M HCl. All tubes were incubated in a shaking boiling water bath (100 °C) for 15 min and cooled at room temperature. Thereafter, n-Butanol (1.5 mL) was added to each tube and thoroughly mixed and then 200 μL of the top phase transferred to a 96- well micro-plate in triplicates and read at 532 and 600 nm using Spectrostar^®^ micro-plate reader. The plasma MDA concentrations were calculated using an extinction coefficient of 1.56 × 10^5^ M^−1^·cm^−1^.

#### 2.4.3. Antioxidant Enzyme Activity

Glutathione peroxidase (GPx) activity in the liver of the rats was determined using a commercially available kit by Cayman chemicals, Ann Arbor, MI, USA. Briefly, 10 mg of liver tissues were homogenized in 90 μL of buffer containing 50 mM Tris-HCl, pH 7.5, 5 mM ethylenediaminetetraacetic acid (EDTA) and 1 mM Dithiothreitol and centrifuged for 15 min at 10,000× *g* at 4 °C. The assay was carried out in a 96-well plate with 20 μL of the supernatant, following the manufacturer’s instructions. GPx activity was subsequently measured as the rate of decrease in absorbance of NADP^+^ at 340 nm on a Spectrostar (Micro-plate reader, Los Angeles, CA, USA).

#### 2.4.4. Liver Carbonyl Protein Determination

This was carried out using a commercial kit (Cayman chemicals, Ann Arbor, MI, USA). Briefly, 100 mg of liver tissues were homogenized in 900 μL of phosphate buffer, pH 6.5, containing EDTA and centrifuged at 10,000× *g* for 15 min at 4 °C. Samples containing 200 μL aliquots each of the supernatant from the liver were placed into two clean glass tubes which served as test and control, respectively. To each tube containing either test or control samples, 800 μL of either 0.2% DNPH or 2.5 M HCl, was added respectively. Samples were then incubated at room temperature in the dark for 1 h with intermittent vortexing and were thereafter treated with either 500 μL of 0.2% DNPH or 500 μL of 2 N HCl, respectively. Protein in both tubes was subsequently precipitated by adding 20% TCA, followed by vigorously mixing the contents of each tube, incubating on ice for 5 min and thereafter spinning the contents of each tube at 10,000× *g* for 10 min at 4 °C. The pellets obtained were further suspended in 10% (*w*/*v*) TCA and incubated on ice for 5 min followed by 10 min centrifugation at 10,000× *g* at 4 °C. The pellets obtained in each case were washed three times in a 1:1 mixture of ethyl acetate and ethanol then resuspended in 6 M guanidine hydrochloride and agitated. The contents (220 μL) of each of test and control tubes were transferred in triplicates into a 96-well microtiter plate and absorbance read at 370 nm using a Spectrostar^®^ micro-plate reader (Los Angeles, CA, USA). An extinction co-efficient value of 0.011 was used in determining the concentration of protein carbonyls in each sample.

#### 2.4.5. Western Blot Detection of Apoptotic Proteins

Protein expression of Bcl-2 associated X protein (Bax) and B-cell lymphoma-2 protein (Bcl-2) were detected using the Western Blot technique. Briefly, 100 mg of liver tissue samples were homogenized in 900 μL of ice-cold radio-immunoprecipitation assay buffer (RIPA buffer) containing 1% protease inhibitor cocktail, 150 mM sodium chloride, 1% triton X-100, 0.5% sodium deoxycholate, 0.1% sodium dodecyl sulphate (SDS) and 50 mM Tris (pH 8) and spun at 12,000 rpm at 4 °C. The resulting supernatant was carefully transferred into fresh pre-cooled tubes and kept on ice and protein content determined using Bradford method [33]. Samples were adjusted for equal loading and 35 μg each of the denatured protein samples were loaded per well and resolved by electrophoresis in a 10% SDS-polyacrylamide gel at 150 mV for 1.5 h at room temperature. Separated proteins were transferred on to a nitrocellulose membrane at 100 mV for 1 h at room temperature, membrane blocked in a 5% bovine serum albumin in Tris-buffered saline (TBS-T) solution for 1 h at room temperature and thereafter incubated overnight at 4 °C in a 1 in 200 dilution of anti-Bax and anti-Bcl primary antibodies raised in rabbit, respectively. Membranes were washed five times in TBS-T solution followed by incubation in a 1 in 1000 dilution of the appropriate horseradish peroxidase conjugated secondary antibodies. Membranes were thereafter washed in TBS-T buffer five times, developed with the Lumiglo reagent (Cell Signaling Technology, Inc., Danvers, MA, USA) and visualized with the ChemiDoc imager (Bio-Rad Laboratories, Inc., Hercules, CA, USA). Image analysis was carried out using the ImageLab^®^ software (Bio-Rad Laboratories, Inc., Hercules, CA, USA).

### 2.5. Electron Microscopy

Glutaraldehyde-fixed samples were washed three times in phosphate buffered saline and post-fixed in 1% osmium tetroxide. Samples were then dehydrated sequentially in 30%, 50%, 70% and 100% acetone solution and left overnight in resin-acetone solution (1:1). Subsequently, samples were transferred into 100% resin for two hours at room temperature and allowed to polymerize in fresh 100% resin solution at 60 °C for eight hours. Using the LEICA EM UC6 (Leica Microsystems GmbH, Wetzlar, Germany) ultramicrotome, 80 microns liver sections were cut, stained with uranyl acetate and lead citrate and subsequently viewed under the JEOL 1010 (Tokyo, Japan) transmission electron microscope (TEM). Micrographs were subsequently analyzed using the iTEM version 5.2 software by two independent observers who were blinded from the study.

### 2.6. Statistical Analysis

All results were expressed as mean ± Standard Error of Mean (S.E.M.). Students’ *t*-test was used to determine statistical differences between groups using the Graph Pad Prism^®^ Software version 5.0 (GraphPad Software, Inc., San Diego, CA, USA). *p* Values less than 0.05 were taken as statistically significant.

## 3. Results

### 3.1. Effects of Naringin on Metabolic Complications of NRTIs

**Figure 1:**
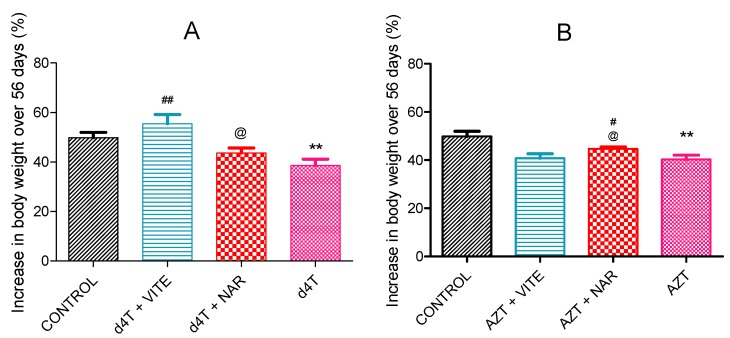
Percentage change in body weight between treatment groups after 56 days of NRTI administration. (**A**) d4T-treated (** *p* < 0.01 compared to control; ## *p* < 0.01 compared to d4T) and (**B**) AZT-treated (** *p* < 0.01 compared to controls; # *p* < 0.05 compared to AZT) rats. @ *p* < 0.05 compared to vitamin E among both d4T and AZT-treated rats.

**Figure 2:**
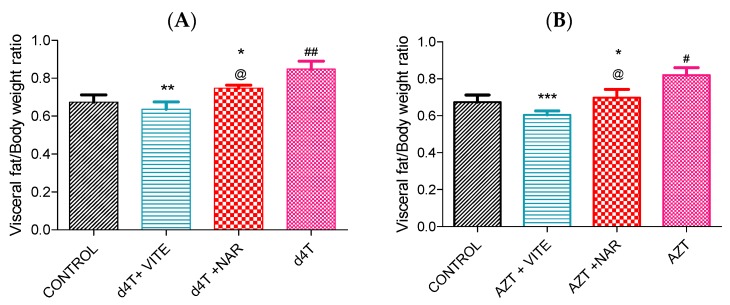
Adiposity index (calculated as a ratio of visceral fat mass to total body weight) among NRTI-treated rats following 56 days of drug treatment. (**A**) d4T-treated (## *p* < 0.01 compared to control; * *p* < 0.05 and ** *p* < 0.01 compared to d4T) and (**B**) AZT-treated (# *p* < 0.05 compared to controls; *** *p* < 0.001 and * *p* < 0.05 compared to AZT) rats. @ *p* < 0.05 compared to vitamin E among both d4T and AZT-treated rats.

**Figure 3:**
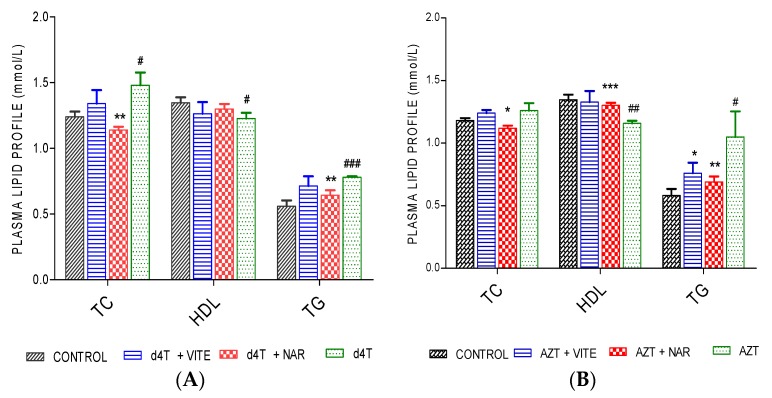
Plasma lipid profile following 56 days of d4T and AZT administration. (**A**) d4T- (** *p* < 0.01 (compared to d4T) and # *p* < 0.05; ### *p* < 0.001 compared to control) and (**B**) AZT- (* *p* < 0.05; ** *p* < 0.01; *** *p* < 0.001 compared to AZT and # *p* < 0.05 and ## *p* < 0.01 compared to control) treated animals after 56 days of drug administrations.

AZT or d4T administration resulted in significant (*p* < 0.05) decrease in total body weight, increase in abdominal fat mass and liver index (calculated as the ratio of the wet liver weight to the total body weight) compared to controls (Figure 1 and Figure 2A,B; Table 2). However, concomitant administration of naringin with either AZT or d4T, led to a significant (*p* < 0.05) increase in the total body weight in the AZT-treated rats (Figure 1B) and a non-significant increase in the d4T-treated rats compared to AZT- or d4T-only treated rats, respectively (Figure 1A). Significant (*p* < 0.05) reduction in abdominal fat mass and liver index were also observed with either vitamin E or naringin treatment compared to AZT- or d4T-only treated rats, respectively (Figure 2A,B; Table 2). Additionally, AZT or d4T caused dyslipidemia evidenced by significant (*p* < 0.05) increases in plasma concentrations of TG and TC and significant (*p* < 0.05) decrease in plasma HDL concentration. Co-administration of either naringin or vitamin E with either AZT or d4T, significantly (*p* < 0.05) reversed dyslipidemia (Figure 3A,B) compared to AZT- or d4T- only treated rats, respectively. The magnitude of the effects of naringin on the afore-mentioned indices of NRTI-induced metabolic complications was similar to those of vitamin E. However, naringin produced a significantly (*p* < 0.05) greater increase in total body weight among the AZT-treated rats compared to vitamin E (Figure 1B), while the reverse was observed in the d4T-treated rats (Figure 1A).

**Table 2:** Liver and total body weight on day 56; apoptotic index (Bax/Bcl-2 ratio).

Parameters	AZT	AZT + NAR	AZT + VITE	d4T	d4T + NAR	d4T + VITE	Control
Final body weight (g)	307.2 ± 3.7 ^a^	316.8 ± 1.99 ^#^	303.2 ± 4.2	303.8 ± 4.1 ^a^	304.2 ± 5.2	315.7 ± 7.5 *	316.6 ± 3.4
Wet liver weight (g)	7.87 ± 0.24 ^aaa^	6.65 ± 0.17 ^###^	6.92 ± 0.20 ^##^	7.134 ± 0.20 ^a^	6.45 ± 0.17 *	6.68 ± 0.21 *	6.57 ± 0.20
Liver index (%)	2.30 ± 0.05 ^a^	2.19 ± 0.02 ^#^	2.44 ± 0.06 ^#^	2.35 ± 0.03 ^a^	2.11 ± 0.07 **	2.31 ± 0.01 *	2.27 ± 0.03
Bax/Bcl-2 ratio	7.20 ± 0.46 ^aaa^	1.18 ± 0.11 ^###^	1.14 ± 0.12 ^###^	5.42 ± 0.23 ^aaa^	1.02 ± 0.05 ***	0.76 ± 0.1 ***	3.18 ± 0.27

Values expressed as mean ± SEM. (^a^ *p* < 0.05 and ^aaa^ *p* < 0.001 compared to control; ^#^ *p* < 0.05; ^##^ *p* < 0.01 and ^###^ *p* < 0.001 compared to zidovudine; * *p* < 0.05; ** *p* < 0.01 and *** *p* < 0.001 compared to stavudine). NAR (naringin) and VITE (vitamin E). (Liver index was calculated as a ratio of wet liver weight to terminal body weight) × 100.

### 3.2. Effects of Naringin on NRTI-induced Oxidative Stress

**Figure 4:**
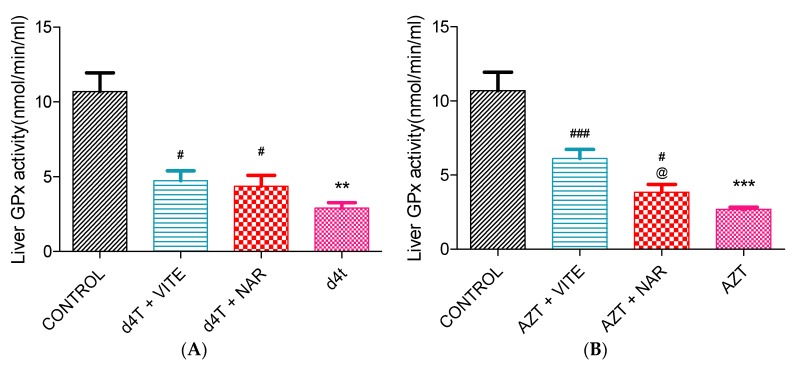
Liver glutathione peroxidase activity following 56 days of drug administration. (**A**) d4T- (** *p* < 0.01 compared to control and # *p* < 0.05 compared to d4T) and (**B**) AZT- (*** *p* < 0.001 compared to control; # *p* < 0.05 and ### *p* < 0.001 compared to AZT) treated animals. @ *p* < 0.05 compared to vitamin E among AZT-treated rats.

**Figure 5:**
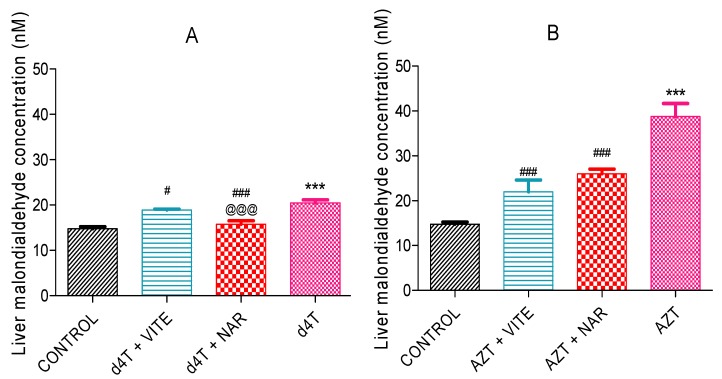
Liver MDA concentrations in: (**A**) d4T- (*** *p* < 0.001 compared to control and # *p* < 0.05; ### *p* < 0.001 compared to d4T and (**B**) AZT- (*** *p* < 0.001 compared to control; ### *p* < 0.001 compared to AZT) treated animals after 56 days of drug administration. @@@ *p* < 0.001 compared to vitamin E among d4T-treated rats.

**Figure 6:**
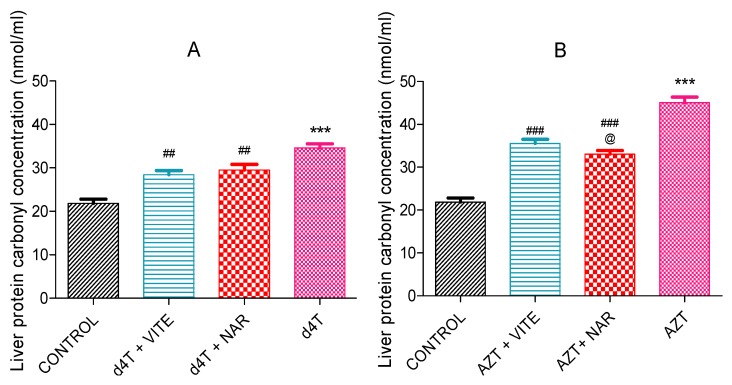
Concentrations of oxidized protein products in the liver after 56 days of drug administration. (**A**) d4T- ((*** *p* < 0.001 compared to control; ## *p* < 0.01 compared to d4T and (**B**) AZT- (*** *p* < 0.001 compared to control; ### *p* < 0.001 compared to AZT) treated animals. @ *p* < 0.05 compared to vitamin E among AZT-treated rats.

AZT- or d4T-only significantly (*p* < 0.05) decreased glutathione peroxidase enzyme activity and significantly (*p* < 0.05) increased concentrations of MDA as well as carbonyl proteins compared to controls. Co-administration of either naringin or vitamin E, however, significantly (*p* < 0.05) increased glutathione peroxidase activity and reduced the concentrations of MDA and carbonyl proteins (Figure 4, Figure 5 and Figure 6A,B) compared to AZT-only and d4T-only treated rats, respectively. While vitamin E produced more significant (*p* < 0.05) improvements in GPx activity among the AZT-treated rats (Figure 4B), naringin appeared to have produced more significant (*p* < 0.05) decreases in MDA and protein carbonyl concentrations among the d4T- and AZT-treated rats, respectively compared to vitamin E (Figure 5A and Figure 6B).

### 3.3. Effects of Naringin on Hepatocyte Apoptosis

**Figure 7:**
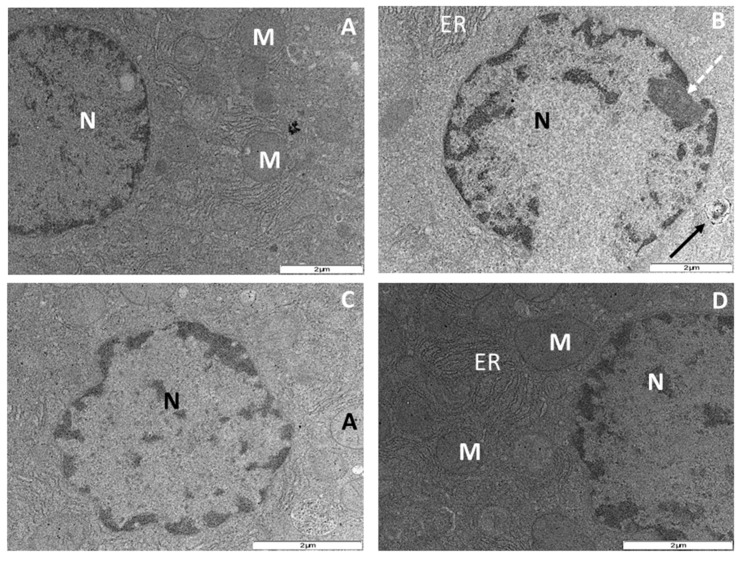
Transmission electron micrograph of rat hepatocytes after 56 days of drug treatment. M = mitochondrion, ER = endoplasmic reticulum and N = nucleus. (**A**) Control rats showing normal nucleus with an even distribution of the nuclear chromatin granules, evenly distributed cytoplasm and preserved mitochondrial architecture; (**B**) AZT-treated rats with markedly reduced mitochondrial population, prominent nucleolus (broken black arrow), clumped and fragmented nuclear chromatin granules coupled with a discontinuation of the nuclear envelope and an apoptotic body (thick back arrow); (**C**) AZT+NAR-treated rats showing minimal clumping of the nuclear chromatin granules, intact nuclear envelope and preserved mitochondrial architecture; (**D**) AZT+VITE-treated rats with intact nuclear envelope, slight clumping of the nuclear chromatin granules in addition to preserved mitochondrial architecture. (Original magnification of reference scale markings: 2 μm = × 15,000).

**Figure 8:**
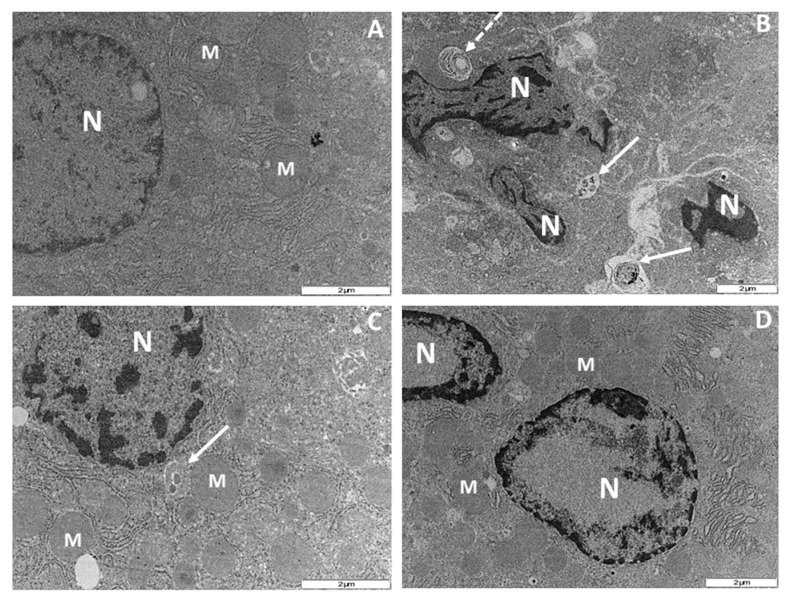
Transmission electron micrograph of rat hepatocytes after 56 days of drug treatment. M = mitochondrion, N = nucleus and ER = endoplasmic reticulum. (**A**) Control rats showing normal nucleus with an even distribution of the nuclear chromatin granules, evenly distributed cytoplasm and preserved mitochondrial architecture; (**B**) d4T-treated rats with severely clumped chromatin granules, broken nuclear membrane and fragmentation of the nucleus. There is an associated condensation of the cytoplasm with appearance of apoptotic bodies (white solid arrows), phagolysosomes (white broken arrows) and sparse mitochondrial population; (**C**) and (**D**) d4T + NAR and d4T + VITE-treated rats, respectively with condensed and fragmented nuclear chromatin granules, numerous mitochondria and even distribution of the cytoplasm. (Original magnification of reference scale markings: 2 μm = × 15,000).

Ultrastructural examination of hepatocytes following 56 days of NRTI therapy revealed apoptotic features induced by administration of AZT- or d4T-only. Treatment with AZT-only resulted in condensation, clumping and fragmentation of nuclear chromatin granules with associated damage to the nuclear envelope compared to the control rats (Figure 7A,B), concomitant administration of naringin or vitamin E moderately reduced the nuclear damage compared to AZT-only treated rats as the nuclear envelopes in both groups of rats remained intact (Figure 7C,D). Additionally, treatment with d4T-only resulted in cytoplasmic condensation, severe clumping and fragmentation of the nuclear chromatin materials, increase in the population of apoptotic bodies and phagolysosmes coupled with reduction in the number of mitochondria in the fields observed (Figure 8A,B). However, co-administration of naringin with d4T minimized nuclear chromatin clumping, nuclear fragmentation and formation of phagolysosmes, maintained mitochondrial population and prevented cytoplasmic condensation (Figure 8C). Similarly, vitamin E prevented cytoplasmic condensation resulting from administration of d4T-only as well as minimized formation of phagolysomes and apoptotic bodies (Figure 8D). These changes were observed to have occurred in a background of intact cellular membrane coupled with preservation of other intracytoplasmic organelles as shown in Figure 7 and Figure 8.

Relative quantification of findings from ultrastructural examination was done with reference to the findings on micrographs from the group of control rats and the average of the readings taken by two independent examiners who were blinded from the study were taken into account (Table 3). Treatment with either AZT- or d4T-only, resulted in increased damage to the nuclear envelope, nuclear fragmentation and nuclear clumping, in addition to increase in the population of phagolysosomes and apoptotic bodies compared to controls. However, co-administration of either naringin or vitamin E caused a relative reduction in damage to the nuclear envelope, nuclear clumping and fragmentation, formation of phagolysosomes and apoptotic bodies which were induced by administration of either NRTI-only.

**Table 3:** Relative quantification of electron microscopy examination of rat hepatocytes.

Treatment Group	Nuclear Clumping and Fragmentation (% of Control)	Damaged Nuclear Membrane (% of Control)	Phagolysosomes (as a Fraction of the Control)	Apoptotic Bodies (as a Fraction of the Control)
AZT only	+++	+	++	+
AZT + NAR	++	-	+	+
AZT + VITE	++	-	+	+
d4T only	+++	++	+++	++
d4T + NAR	++	-	+	+
d4T + VITE	++	-	+	+
CONTROL	-	-	-	-

+++ ≥ 50%, ++ = 20% to 50% and + ≤ 20% relative to control rats. Values recorded are an average of the values obtained by observation of at least five fields from the same sample by two independent examiners who were blinded to the experiment carried out.

**Figure 9:**
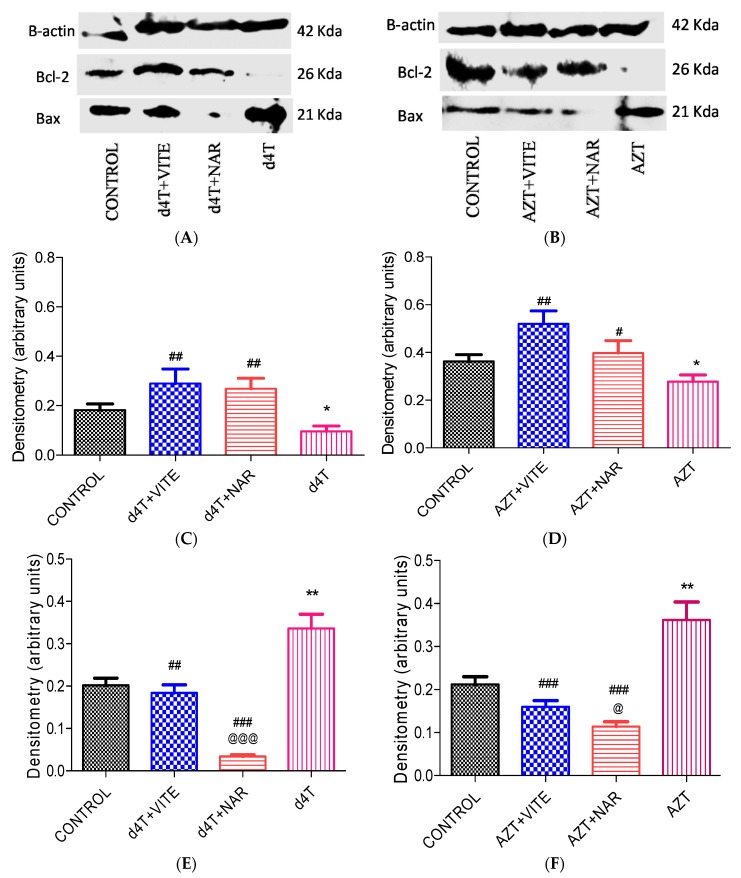
Bax and Bcl-2 protein expression following 56 days of NRTI treatment. (**A**) and (**B**) show Bax, Bcl-2 and beta actin protein expression whilst (**C**), (**D**), (**E**) and (**F**) show the densitometry scans of the respective proteins normalized to the house-keeping protein (beta actin) following 56 days of drug administration; (**C**) and (**E**) d4T- (* *p* < 0.05; ** *p* < 0.01 compared to control and ## *p* < 0.01; ### *p* < 0.001 compared to d4T); (**D**) and (**F**) AZT- (* *p* < 0.05; ** *p* < 0.01 compared to control and # *p* < 0.05; ## *p* < 0.01; ### *p* < 0.001 compared to AZT). @@@ *p* < 0.001 and @ *p* < 0.05 compared to vitamin E in d4T and AZT-treated rats, respectively.

Furthermore, there was significantly (*p* < 0.05) increased expression of Bcl-2 protein and decreased Bax protein expression with co-administration of either naringin or vitamin E with either of the NRTIs (Figure 9). The effect of naringin on Bcl-2 protein expression among d4T- and AZT-treated rats was comparable to the effects of vitamin E (Figure 9C,D). Naringin, however, appeared to have produced a more significant (*p* < 0.05) reduction in the expression of Bax protein compared to vitamin E in AZT- and d4T-treated groups of rats where either naringin or vitamin E was co-administered (Figure 9E,F), respectively. Furthermore, significant (*p* < 0.001) increases in Bax/Bcl-2 ratio arising from AZT or d4T treatment were significantly (*p* < 0.001) reversed by concomitant administration of either naringin or vitamin E (Table 2) with AZT or d4T, respectively.

## 4. Discussion

Known metabolic complications of NRTI administration include lipodystrophy, dyslipidemia, hepatotoxicity, hepatomegaly, metabolic syndrome, hyperlactatemia, and cardiomyopathy [11,13,34,35,36]. Cellular oxidative damage caused by mitochondrial toxicity is one of the numerous scientific mechanisms that underscore the development of these complications [22,37]. Common antioxidants such as vitamins C and E, uridine as well as carnitine have been investigated in preventing or reversing these complications with minimal success [38,39,40]. Therefore, further screening of newer and perhaps more efficacious antioxidants in managing these complications becomes necessary. Naringin is a readily available and cheap dietary flavonoid present in most citrus fruits with proven antioxidant and anti-apoptotic properties which have been demonstrated in *in vitro*, *in vivo* and *ex vivo* animal models [41,42,43]. Its candidacy in the management of NRTI-induced metabolic complications is worth investigating.

In this study, we established a model of NRTI-induced metabolic complications, investigated possible mechanisms involved and probed the usefulness of naringin, compared to vitamin E, in ameliorating these complications. Presence of lipodystrophy, evidenced by significant increase in the adiposity index (Figure 2), dyslipidemia (Figure 3) and hepatic enlargement (Table 2), in the presence of significantly reduced total body weight (Table 1) in AZT or d4T-treated rats, were taken as markers of NRTI-induced metabolic complications [44,45,46]. At the doses administered in this present study, AZT and d4T have previously been shown to exert toxic effects ranging from steatosis, hyperbilirubinemia, hypoproteinemia, ultrastructural damage to the liver as well as the neurons, and oxidative stress [27,29]. Co-administration of naringin with either AZT or d4T, significantly reversed these metabolic complications similarly to vitamin E, as evidenced by significant improvements in the total body weight, reduction of the hypertriglyceridemia and hypercholesterolemia as well as increasing plasma HDL concentrations.

An imbalance between the production of reactive oxygen species (ROS) and intracellular antioxidant capacity underlies the development of oxidative injury which forms the basis for the development of many pathologic conditions [47]. A decrease or an increase in the activities of enzymatic antioxidant proteins (manganese superoxide dismutase (MnSOD) and GPx) has consistently been noted during oxidative stress [48,49]. An unchecked increase in ROS within the cell eventually results in lipid peroxidation, oxidative protein and nucleic acid damage [50,51]. These damages to the cellular framework ultimately cascades into inhibition of cellular enzyme activity and activation of the mechanisms for programmed cell death which eventually leads to cellular demise [50,51,52]. In this study, NRTI-treated rats exhibited significant increases in MDA (Figure 5) and carbonyl proteins (end products of intracellular oxidative damage to lipids and proteins) (Figure 6) concentrations similar to the findings of Banerjee *et al*. [16]. These were observed against a background of a significant decrease in the activity of glutathione peroxidase. Increased oxidative stress can lead to a reduction in antioxidant enzyme activity due to reduction in the gene expression or suppression of antioxidant proteins’ production by oxidative stress [40]. NRTIs have been shown to cause a reduction in antioxidant protein gene expression [21]. A picture of significantly reduced glutathione peroxidase activity (Figure 4) coupled with significantly raised MDA (Figure 5) and carbonyl proteins concentration (Figure 6), suggests a state of overwhelming oxidative stress following NRTI treatment which was significantly improved by concomitant administration of either naringin or vitamin E with AZT or d4T, respectively. Naringin, as an antioxidant, has previously been shown to improve antioxidant gene expression and antioxidant enzyme activity at the dose it was administered in the present study [41,53,54]. On the other hand, vitamin E has previously been administered at various doses for its ability to prevent lipid peroxidation in various tissues [31,55,56].

Pro-apoptotic effects of NRTIs have also been demonstrated previously [35,57]. Increased Bax protein expression, reduced Bcl-2 protein expression, increased Bax/Bcl-2 ratio, and ultrastructural apoptotic changes such as karyorrhexis, karyolysis and formation of apoptotic bodies, in a background of preserved architecture of other intracellular organelles, have been used as markers of apoptosis [58,59,60]. Furthermore, electron microscopy findings of these ultrastructural changes are regarded as the “gold standard” in identifying apoptosis [61]. In the present study, naringin was observed to have mitigated AZT or d4T-induced apoptosis within the liver tissue comparably to vitamin E as evidenced by a significant reduction in the expression of the pro-apoptotic protein Bax (Figure 9E,F) and a significant increase in the expression of the anti-apoptotic protein Bcl-2 (Figure 9C,D). Bax is a cytosolic protein, which when activated by apoptotic triggers such as ROS [62], translocates to the outer mitochondrial membrane. This causes a reduction in the mitochondrial membrane potential, mitochondrial membrane leakage, activation of caspases and other pro-apoptotic agents, leading to an increase in the rate of apoptosis within the tissue. Bcl-2 on the other hand, prevents apoptosis by binding to and inhibiting pro-apoptotic proteins such as Bcl-2 homology domain 3 protein (BH3) [63]. Antioxidant intake has been associated with an altered rate of cellular death [32] and indeed naringin has previously been shown to possess anti-apoptotic properties [41,64]. Apoptosis plays an important role in the development of some of these metabolic complications of NRTIs and amelioration of the same is required to minimize these complications. From the present study, naringin appeared to have minimized the metabolic complications of NRTIs in a similar fashion to vitamin E’s effects in addition to reducing oxidative stress and apoptotic changes. This finding therefore suggests that naringin may be beneficial in such cases of NRTI-induced complications wherein oxidative stress and apoptosis play important roles in their pathogenesis. Furthermore, in d4T-treated rats, we observed the presence of phagocytic lysosomes (a marker of an ongoing autophagic process) [65]. Conversely, naringin or vitamin E co-treatment appear to reduce the development of phagolysosomes and ultrastructural changes indicative of apoptosis, thus further lending credence to the anti-apoptotic effects of naringin. Our study therefore suggests that co-administration of naringin, a dietary flavonoid, together with NRTIs, mitigates AZT or d4T-induced metabolic complications, oxidant stress, apoptosis and autophagy similarly to vitamin E.

Although this study provides preliminary evidence of potential amelioration of metabolic complications of NRTIs, by naringin, it is not clear whether naringin’s effects are due to its action on mitochondrial structural defects and function and further investigation of mitochondrial morphology and function is needed. Mitochondrial population reduction, cristae fragmentation, lamellar degeneration, swelling and outer membrane disruptions are some of the reported features of NRTI-induced ultrastructural changes that might contribute to mitochondrial dysfunction and metabolic complications of NRTIs [66,67]. Furthermore, NRTI-induced mitochondrial toxicity is suggested to underlie the development of metabolic complications associated with the use of these drugs [22]. Therefore, a thorough investigation of mitochondrial ATP generation, lactate levels, mitochondrial calcium concentration and specific markers of mitochondrial dysfunction such as uncoupling proteins (UCP-2) and mtDNA depletion and/or mutation would provide a better understanding of naringin’s mechanism of ameliorating NRTI-induced metabolic complications. Moreover, the contribution of endoplasmic reticulum (ER) stress to mitochondrial dysfunction has recently become apparent and it is currently believed that the ER and mitochondria are structurally and functionally related [68,69]. Therefore, a dissection of NRTI effects on ER stress in relation to mitochondrial dysfunction might have provided us with a deeper insight into the role of naringin in alleviating metabolic complications of NRTIs. Therefore, future studies would endeavor to look at these parameters in order to provide a fuller understanding of the role of naringin in alleviating NRTI-induced metabolic complications.

## 5. Conclusions

Naringin’s reversal of some of the NRTI-induced metabolic complications provides preliminary evidence of its potential in mitigating NRTI-induced metabolic complications. The mechanism by which naringin ameliorate these metabolic complication possibly involves its antioxidant and/or anti-apoptotic effects. However, a better understanding of its role in the pathophysiology of NRTI-induced metabolic complications and mitochondrial dysfunction needs to be further evaluated.

## References

[1] Panos G., Samonis G., Alexiou V.G., Kavarnou G.A., Charatsis G., Falagas M.E. (2008). Mortality and morbidity of HIV infected patients receiving HAART: A cohort study. Curr. HIV Res..

[2] Sabin C.A., Smith C.J., Youle M., Lampe F.C., Bell D.R., Puradiredja D., Lipman M.C., Bhagani S., Phillips A.N., Johnson M.A. (2006). Deaths in the era of HAART: Contribution of late presentation, treatment exposure, resistance and abnormal laboratory markers. AIDS.

[3] Yang C.H., Huang Y.F., Hsiao C.F., Yeh Y.L., Liou H.R., Hung C.C., Yang S.Y. (2008). Trends of mortality and causes of death among HIV-infected patients in Taiwan, 1984–2005. HIV Med..

[4] Hoschele D. (2006). Cell culture models for the investigation of NRTI-induced mitochondrial toxicity. Relevance for the prediction of clinical toxicity. Toxicol. Vitro.

[5] Thompson M.A., Aberg J.A., Cahn P., Montaner J.S., Rizzardini G., Telenti A., Gatell J.M., Günthard H.F., Hammer S.M., Hirsch M.S. (2010). Antiretroviral treatment of adult HIV infection: 2010 recommendations of the International AIDS Society-USA panel. JAMA.

[6] Patel K., van Dyke R.B., Mittleman M.A., Colan S.D., Oleske J.M., Seage G.R. (2012). The impact of HAART on cardiomyopathy among children and adolescents perinatally infected with HIV-1. AIDS.

[7] Morris K. (1998). Short course of AZT halves HIV-1 perinatal transmission. Lancet.

[8] Senise J.F., Castelo A., Martínez M. (2011). Current treatment strategies, complications and considerations for the use of HIV antiretroviral therapy during pregnancy. AIDS Rev..

[9] Palmer M., Chersich M., Moultrie H., Kuhn L., Fairlie L., Meyers T. (2013). Frequency of stavudine substitution due to toxicity in children receiving antiretroviral treatment in sub-Saharan Africa. AIDS.

[10] Maskew M., Westreich D., Fox M.P., Maotoe T., Sanne I.M. (2012). Effectiveness and safety of 30 mg *versus* 40 mg stavudine regimens: A cohort study among HIV-infected adults initiating HAART in South Africa. J. Int. AIDS Soc..

[11] Cabrero E., Griffa L., Burgos A. (2010). Prevalence and impact of body physical changes in HIV patients treated with highly active antiretroviral therapy: Results from a study on patient and physician perceptions. AIDS Patient Care STDs.

[12] Calza L., Manfredi R., Chiodo F. (2005). Hyperlactataemia and lactic acidosis in HIV-infected patients receiving antiretroviral therapy. Clin. Nutr..

[13] Wierzbicki A.S., Purdon S.D., Hardman T.C., Kulasegaram R., Peters B.S. (2008). HIV lipodystrophy and its metabolic consequences: Implications for clinical practice. Curr. Med. Res. Opin..

[14] Hammond E., McKinnon E., Nolan D. (2010). Human immunodeficiency virus treatment-induced adipose tissue pathology and lipoatrophy: Prevalence and metabolic consequences. Clin. Infect. Dis..

[15] Fiorenza C.G., Chou S.H., Mantzoros C.S. (2011). Lipodystrophy: Pathophysiology and advances in treatment. Nat. Rev. Endocrinol..

[16] Banerjee A., Abdelmegeed M.A., Jang S., Song B. (2013). Zidovudine (AZT) and hepatic lipid accumulation: Implication of inflammation, oxidative and endoplasmic reticulum stress mediators. PLoS ONE.

[17] Sutinen J., Häkkinen A.M., Westerbacka J., Seppälä-Lindroos A., Vehkavaara S., Halavaara J., Järvinen A., Ristola M., Yki-Järvinen H. (2002). Increased fat accumulation in the liver in HIV-infected patients with antiretroviral therapy-associated lipodystrophy. AIDS.

[18] World Health Organization (2014). March 2014 Supplement to the 2013 Consolidated Guidelines on the Use of Antiretroviral Drugs for Treating and Preventing HIV Infection—Recommendations for a Public Health Approach.

[19] Apostolova N., Blas-Garcia A., Esplugues J.V. (2011). Mitochondrial interference by anti-HIV drugs: Mechanisms beyond Pol-gamma inhibition. Trends Pharmacol. Sci..

[20] Fang J., Beland F.A. (2009). Long-Term exposure to zidovudine delays cell cycle progression, induces apoptosis, and decreases telomerase activity in human hepatocytes. Toxicol. Sci..

[21] Prakash O., Teng S., Ali M., Zhu X., Coleman R., Dabdoub R.A., Chambers R., Aw T.Y., Flores S.C., Joshi B.H. (1997). The human immunodeficiency virus type 1 Tat protein potentiates zidovudine-induced cellular toxicity in transgenic mice. Arch. Biochem. Biophys..

[22] Perez-Matute P., Pérez-Martínez L., Blanco J.R., Oteo J.A. (2013). Role of mitochondria in HIV infection and associated metabolic disorders: Focus on nonalcoholic fatty liver disease and lipodystrophy syndrome. Oxid. Med. Cell. Longev..

[23] Wanchu A., Rana S.V., Pallikkuth S., Sachdeva R.K. (2009). Short communication: Oxidative stress in HIV-infected individuals: A cross-sectional study. AIDS Res. Hum. Retrovir..

[24] Rajadurai M., Prince P.S.M. (2006). Preventive effect of naringin on lipid peroxides and antioxidants in isoproterenol-induced cardiotoxicity in Wistar rats: Biochemical and histopathological evidences. Toxicology.

[25] Chanet A., Milenkovic D., Manach C., Mazur A., Morand C. (2012). Citrus flavanones: What is their role in cardiovascular protection?. J. Agric. Food Chem..

[26] Benavente-García O., Castillo J. (2008). Update on uses and properties of citrus flavonoids: New findings in anticancer, cardiovascular, and anti-inflammatory activity. J. Agric. Food Chem..

[27] Nayak A., Singh M., Mishra A. (2013). Hepatotoxic changes induced by prenatal administration of zidovudine in Swiss albino mice. Int. J. Ther. Appl..

[28] Tortorella C., Guidolin D., Petrelli L., de Toni R., Milanesi O., Ruga E., Rebuffat P., Bova S. (2009). Prolonged zidovudine administration induces a moderate increase in the growth and steroidogenic capacity of the rat adrenal cortex. Int. J. Mol. Med..

[29] Weber J., Mitchell D., Kamerman P.R. (2007). Oral administration of stavudine induces hyperalgesia without affecting activity in rats. Physiol. Behav..

[30] Xulu S., Owira P.M.O. (2012). Naringin ameliorates atherogenic dyslipidemia but not hyperglycemia in rats with type 1 diabetes. J. Cardiovasc. Pharmacol..

[31] Ishaq G.M., Saidu Y., Bilbis L.S., Muhammad S.A., Jinjir N., Shehu B.B. (2013). Effects of α-tocopherol and ascorbic acid in the severity and management of traumatic brain injury in albino rats. J. Neurosci. Rural Pract..

[32] Halliwell B., Chirico S. (1993). Lipid peroxidation: Its mechanism, measurement, and significance. Am. J. Clin. Nutr..

[33] Bradford M.M. (1976). A dye binding assay for protein. Anal. Biochem..

[34] Calza L., Manfredi R., Colangeli V., Tampellini L., Sebastiani T., Pocaterra D., Chiodo F. (2005). Substitution of nevirapine or efavirenz for protease inhibitor *versus* lipid-lowering therapy for the management of dyslipidaemia. AIDS.

[35] Kinpara S., Kijiyama M., Takamori A., Hasegawa A., Sasada A., Masuda T., Tanaka Y., Utsunomiya A., Kannagi M. (2013). Interferon-α (IFN-α) suppresses HTLV-1 gene expression and cell cycling, while IFN-α combined with zidovudin induces p53 signaling and apoptosis in HTLV-1-infected cells. Retrovirology.

[36] Vigano A., Brambilla P., Cafarelli L., Giacomet V., Borgonovo S., Zamproni I., Zuccotti G., Mora S. (2007). Normalization of fat accrual in lipoatrophic, HIV-infected children switched from stavudine to tenofovir and from protease inhibitor to efavirenz. Antiviral Ther..

[37] Jiang B., Khandelwal A.R., Rogers L.K., Hebert V.Y., Kleinedler J.J., Zavecz J.H., Shi W., Orr A.W., Dugas T.R. (2010). Antiretrovirals induce endothelial dysfunction via an oxidant-dependent pathway and promote neointimal hyperplasia. Toxicol. Sci..

[38] Walker U.A., Venhoff N. (2005). Uridine in the prevention and treatment of NRTI-related mitochondrial toxicity. Antiviral Ther..

[39] Lebrecht D., Deveaud C., Beauvoit B., Bonnet J., Kirschner J., Walker U.A. (2008). Uridine supplementation antagonizes zidovudine-induced mitochondrial myopathy and hyperlactatemia in mice. Arthritis Rheum..

[40] Borut P., Dušan Š., Irina M. (2013). Achieving the balance between ROS and antioxidants: When to use the synthetic antioxidants. Oxid. Med. Cell. Longev..

[41] Bharti S., Rani N., Krishnamurthy B., Arya D.S. (2014). Preclinical evidence for the pharmacological actions of Naringin: A review. Planta Med..

[42] Chen J., Guo R., Yan H., Tian L., You Q., Li S.Q., Huang R., Wu K. (2014). Naringin inhibits ROS-activated MAPK pathway in high glucose-induced injuries in H9c2 cardiac cells. Basic Clin. Pharmacol. Toxicol..

[43] Ikemura M., Sasaki Y., Giddings J.C., Yamamoto J. (2012). Preventive effects of hesperidin, glucosyl hesperidin and naringin on hypertension and cerebral thrombosis in stroke-prone spontaneously hypertensive rats. Phytother. Res..

[44] Fernandez C.D.B., Bellentani F.F., Fernandes G.S.A., Perobelli J.E., Favareto A.P.A., Nascimento A.F., Cicogna A.C., Kempinas W.D. (2011). Diet-induced obesity in rats leads to a decrease in sperm motility. Reprod. Biol. Endocrinol..

[45] Prasad S.S., Prashanth A., Kumar C.P., Reddy S.J., Giridharan N.V., Vajreswari A. (2010). A novel genetically-obese rat model with elevated 11beta-hydroxysteroid dehydrogenase type 1 activity in subcutaneous adipose tissue. Lipids Health Dis..

[46] Günthard H.F., Aberg J.A., Eron J.J., Hoy J.F., Telenti A., Benson C.A., Burger D.M., Cahn P., Gallant J.E., Glesby M.J. (2014). Antiretroviral treatment of adult HIV infection: 2014 recommendations of the International Antiviral Society-USA Panel. JAMA.

[47] Lobo V., Patil A., Phatak A., Chandra N. (2010). Free radicals, antioxidants and functional foods: Impact on human health. Pharmacog. Rev..

[48] Afolayan A.J., Eis A., Teng R., Bakhutashvili I., Kaul S., Davis J.M., Konduri G.G. (2012). Decreases in manganese superoxide dismutase expression and activity contribute to oxidative stress in persistent pulmonary hypertension of the newborn. Am. J. Physiol. Lung Cell. Mol. Physiol..

[49] Goyal R., Singhai M., Faizy A.F. (2011). Glutathione peroxidase activity in obese and nonobese diabetic patients and role of hyperglycemia in oxidative stress. J. Midlife Health.

[50] Sharma P., Jha A.B., Dubey R.S., Pessarakli M. (2012). Reactive oxygen species, oxidative damage, and antioxidative defense mechanism in plants under stressful conditions. J. Bot..

[51] Srivastava S., Dubey R.S. (2011). Manganese-excess induces oxidative stress, lowers the pool of antioxidants and elevates activities of key antioxidative enzymes in rice seedlings. Plant Growth Regul..

[52] Mishra S., Jha A.B., Dubey R.S. (2011). Arsenite treatment induces oxidative stress, upregulates antioxidant system, and causes phytochelatin synthesis in rice seedlings. Protoplasma.

[53] Jeon S.M., Bok S.H., Jang M.K., Lee M.K., Nam K.T., Park Y.B., Rhee S.J., Choi M.S. (2001). Antioxidative activity of naringin and lovastatin in high cholesterol-fed rabbits. Life Sci..

[54] Bakheet S.A., Attia S.M. (2011). Evaluation of chromosomal instability in diabetic rats treated with naringin. Oxid. Med. Cell. Longev..

[55] Cristina D.C., del Rosario R.M., Rosa A.C., Veronica O.A. (2013). On the performance of trimetazidine and vitamin E as pharmacoprotection agents in cyclosporin A-induced toxicity. ISRN Pharmacol..

[56] Brinkmann K., ter Hofstede H.J.M. (1999). Mitochondrial toxicity of nucleoside analogue reverse transcriptase inhibitors: Lactic acidosis, risk factors and therapeutic options. AIDS Rev..

[57] Caron M., Auclair M., Lagathu C., Lombès A., Walker U.A., Kornprobst M., Capeau J. (2004). The HIV-1 nucleoside reverse transcriptase inhibitors stavudine and zidovudine alter adipocyte functions *in vitro*. AIDS.

[58] Woo-Sung M., Joo-Heon K., Myoung-Jae K., Dong-Geun L. (2000). Early ultrastructural changes of apoptosis induced by fumonisin B1 in rat liver. Yonsei Med. J..

[59] Salakou S., Kardamakis D., Tsamandas A.C., Zolota V., Apostolakis E., Tzelepi V., Papathanasopoulos P., Bonikos D.S., Papapetropoulos T., Petsas T. (2007). Increased Bax/Bcl-2 ratio up-regulates caspase-3 and increases apoptosis in the thymus of patients with myasthenia gravis. Vivo.

[60] Kwong J., Choi H.L., Huang H., Chan F.L. (1999). Ultrastructural and biochemical observations on the early changes in apoptotic epithelial cells of the rat prostate induced by castration. Cell Tissue Res..

[61] Taatjes D.J., Sobel B.E., Budd C. (2008). Morphological and cytochemical determination of cell death by apoptosis. Histochem. Cell Biol..

[62] Wen J., You K., Lee S., Song C., Kim D. (2002). Oxidative stress-mediated apoptosis: The anticancer effect of the sesquiterpene lactone parthenolide. J. Biol. Chem..

[63] Shamas-Din A., Kale J., Leber B., Andrews D.W. (2013). Mechanisms of action of Bcl-2 family proteins. Cold Spring Harb. Perspect. Biol..

[64] Kandhare A.D., Ghosh P., Bodhankar S.L. (2014). Naringin, a flavanone glycoside, promotes angiogenesis and inhibits endothelial apoptosis through modulation of inflammatory and growth factor expression in diabetic foot ulcer in rats. Chem. Biol. Interact..

[65] Amaravadi R.K., Thompson C.B. (2007). The roles of therapy-induced autophagy and necrosis in cancer treatment. Clin. Cancer Res..

[66] D'Amati G., Kwan W., Lewis W. (1992). Dilated cardiomyopathy in a zidovudine-treated AIDS patient. Cardiovasc. Pathol..

[67] Pezeshkpour G., Illa I., Dalakas M.C. (1992). Ultrastructural characteristics and DNA immunocytochemistry in human immunodeficiency virus and zidovudine-associated myopathies. Hum. Pathol..

[68] Rutter G.A., Pinton P. (2014). Mitochondria-associated endoplasmic reticulum membranes in insulin signaling. Diabetes.

[69] Santulli G., Pagano G., Sardu C., Xie W., Reiken S., D’Ascia S.L., Cannone M., Marziliano N., Trimarco B., Guise T.A. (2015). Calcium release channel RyR2 regulates insulin release and glucose homeostasis. J. Clin. Investig..

